# Phytochemical profile and antifungal activity of essential oils obtained from different *Mentha longifolia* L. accessions growing wild in Iran and Iraq

**DOI:** 10.1186/s12870-024-05135-z

**Published:** 2024-05-27

**Authors:** Kosrat Hama Mustafa, Jalal Khorshidi, Yavar Vafaee, Azad Rastegar, Mohammad Reza Morshedloo, Somaieh Hossaini

**Affiliations:** 1 Department of Horticultural Science and Engineering, Faculty of Agriculture, University of Kurdistan, Sanandaj, 66177-15175 Iran; 2 Department of Horticulture, College of Agricultural Engineering Sciences, University of Raparin, Ranya, Iraq; 3 Research Center of Medicinal Plants Breeding and Development, University of Kurdistan, Sanandaj, Iran; 4 Forests and Rangelands Research Department, Kurdistan Agricultural and Natural Resources Research and Education Center, Sanandaj, Iran; 5 Department of Horticultural Science and Engineering, Faculty of Agriculture, University of Maragheh, Maragheh, Iran; 6 Department of Plant Protection, Faculty of Agriculture, University of Kurdistan, Sanandaj, 66177-15175 Iran

**Keywords:** Biological activity, Chemical composition, Fumigation, *Fusarium solani*

## Abstract

**Background:**

*Mentha longifolia* L. is a perennial plant belonging to the Lamiaceae family that has a wide distribution in the world. *M. longifolia* has many applications in the food and pharmaceutical industries due to its terpenoid and phenolic compounds. The phytochemical profile and biological activity of plants are affected by their genetics and habitat conditions. In the present study, the content, constituents and antifungal activity of the essential oil extracted from 20 accessions of *M. longifolia* collected from different regions of Iran and Iraq countries were evaluated.

**Results:**

The essential oil content of the accessions varied between 1.54 ± 0.09% (in the Divandarreh accession) to 5.49 ± 0.12% (in the Khabat accession). Twenty-seven compounds were identified in the essential oils of the studied accessions, which accounted for 85.5-99.61% of the essential oil. The type and amount of dominant compounds in the essential oil were different depending on the accession. Cluster analysis of accessions based on essential oil compounds grouped them into three clusters. The first cluster included Baziyan, Boukan, Sarouchavah, Taghtagh, Darbandikhan, Isiveh and Harir. The second cluster included Khabat, Kounamasi, Soni and Mahabad, and other accessions were included in the third cluster. Significant correlations were observed between the essential oil content and components with the climatic and soil conditions of the habitats. The *M. longifolia* essential oil indicated antifungal activity against *Fusarium solani* in both methods used. In all studied accessions, the fumigation method compared to the contact method was more able to control mycelia growth. In both methods, the inhibition percentage of essential oil on mycelia growth increased with an increase in essential oil concentration. Significant correlations were found between the essential oil components and the inhibition percentage of mycelium growth.

**Conclusion:**

The studied *M. longifolia* accessions showed significant differences in terms of the essential oil content and components. Differences in phytochemical profile of accessions can be due to their genetic or habitat conditions. The distance of the accessions in the cluster was not in accordance with their geographical distance, which indicates the more important role of genetic factors compared to habitat conditions in separating accessions. The antifungal activity of essential oils was strongly influenced by the essential oil quality and concentration, as well as the application method. Determining and introducing the elite accession in this study can be different depending on the breeder’s aims, such as essential oil content, desired chemical composition, or antifungal activity.

## Introduction

*Mentha longifolia* L. (horsemint) is a perennial plant belonging to the Lamiaceae family which is distributed in temperate and semi-tropical regions of Europe, west and south of Asia, and north and south of Africa [1]. In the food industry, *M. longifolia* is used in the form of raw or processed as a spice or preservative [2]. In traditional medicine, this plant is used to treat various diseases such as common cold, cough, sore throat, fever, nausea, nasal congestion, diarrhea, gut spasm, sinusitis, bronchitis, indigestion, intestinal colic, liver disorders and general weakness. Also, this plant is known as a sedative, appetizer, anti-pruritic and insect repellent [3–9]. Anti-inflammatory [10], antioxidant [11], anti-mutation [12], anti-cancer [13], antibacterial [14], antifungal [11], and antiviral [15] properties of *M. longifolia* have been proven in previous studies. Phenolic acids, flavonoids and terpenes are the most important effective substances of *M. longifolia* [8]. Many studies have been conducted on *M. longifolia* essential oil, the results of which indicate a remarkable diversity in its content and constituents [16–23]. Pathogenic fungi are one of the most destructive microorganisms that cause major damage to agricultural crops before or after harvest [24]. One of the most well-known pathogenic fungi is *Fusarium solani*. This fungus species has more than 100 hosts among agricultural crops and typically causes crown and root rot in host plants. The infected plants show wilting, stunting, chlorosis, and stem lesions [25]. There are various agricultural, chemical and biological methods to control fungal diseases, but chemical poisons are usually used. The use of chemical poisons to control pathogenic fungi should be limited due to their harmful effects on humans and the environment [26]. The effective compounds of plants, such as essential oils, are a suitable choice to replace chemical poisons due to their high antifungal potential [27]. The antifungal potential of *M. longifolia* essential oil has been proven in many studies [28–33]. The antifungal activity of plant essential oils can vary depending on the type and amount of essential oil constituents [34]. The phytochemical profile of plants is influenced by the genetics and ecological conditions of their habitat. Different accessions belonging to a plant species, due to their different genetics and ecological conditions, have different phytochemical profiles and biological activities. This study aimed to evaluate the phytochemical profile and antifungal activity of essential oils obtained from different *M. longifolia* accessions collected from Iran and Iraq to identify the superior accession as well as the optimal ecological conditions for the production of essential oil with the desired quantity and quality.

## Materials and methods

### Identification of *M. longifolia* habitats, and plant and soil sampling

**Fig. 1:**
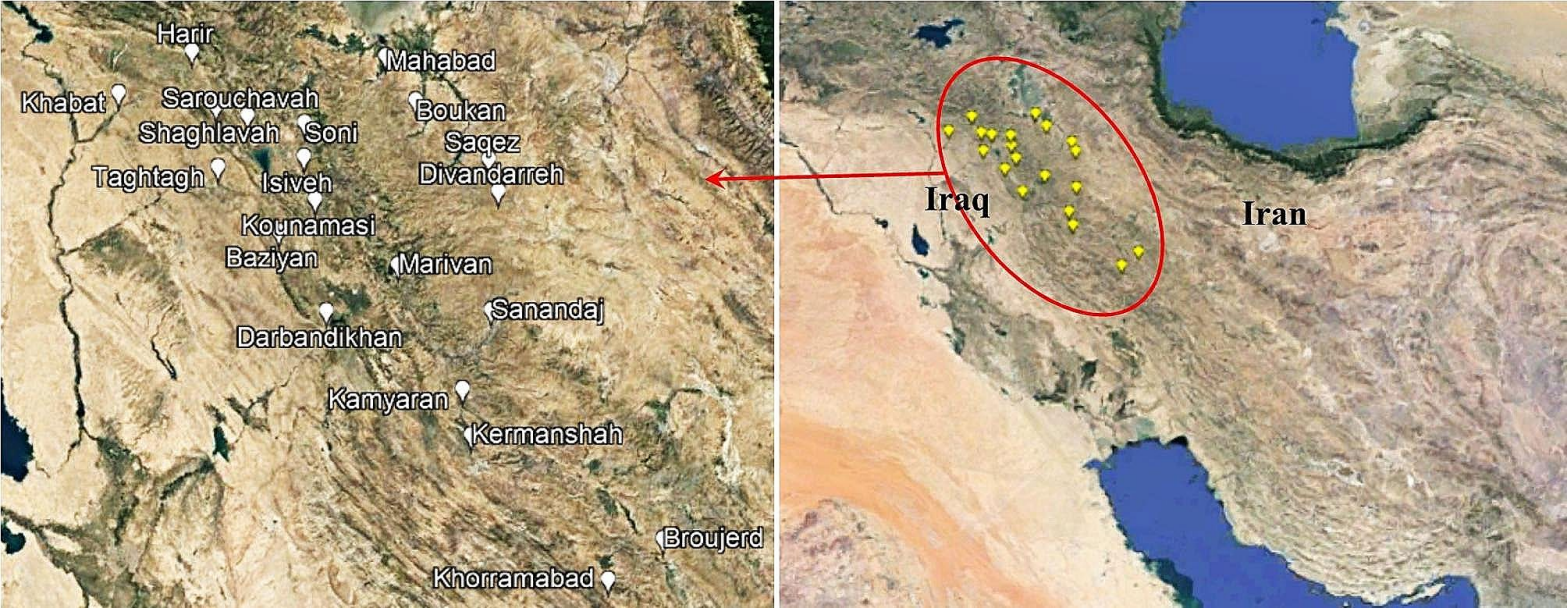
Geographical location of the studied *M. longifolia* accessions

First, with the help of reliable sources [35] and the information of local people, the habitats of *M. longifolia* were identified in the northwest and west of Iran, and the east of Iraq (Fig. 1). Then, at the full flowering stage (early July 2021), the habitats were visited, and ten *M. longifolia* samples were collected from each habitat. The collected plant samples were dried in the shade and prepared for essential oil extraction. A voucher specimen of *M. longifolia* (HKM2361) identified by Dr. Azad Rastegar has been deposited in the HKS herbarium of Agriculture and Natural Resources Research Center, Sanandaj, Iran. Simultaneously with the plant sampling, one soil sample was prepared from each habitat for physicochemical analysis. The results of soil analysis are shown in Table 1. The geographical coordinates of habitats were obtained by GPS device and Google Earth software. Average monthly temperature and average monthly rainfall of habitats were obtained from the closest weather station to each habitat (Table 1).

### Essential oil extraction

The essential oils from plant samples were extracted by Clevenger apparatus based on the hydro distillation method. The essential oil extraction was performed for three hours with three repetitions for each accession. After the essential oil extraction, the volume of the collected essential oil was measured, and its v/w % was calculated. Then, the essential oil was separated from the Clevenger and poured into a glass vial. The obtained essential oils were dehydrated with anhydrous sodium sulfate and kept in the refrigerator at 4 °C until analysis.

### Essential oil analysis

GC-MS and GC-FID were used for essential oil analysis. The device’s specifications and their working conditions are given below.

GC-MS: Agilent 7890B/5977A GC/MSD with HP-5 capillary column (30 m length, 0.25 mm inner diameter, 0.25 μm film thickness) was used to identify compounds. The injection chamber temperature was set at 280 °C. The initial temperature of the column was 50 °C for 1 min, and then raised to 260 °C at a rate of 10 °C/min. Helium was used as a carrier gas at a flow rate of 1 mL/min; ionization source temperature, 230 °C; ionization energy, 70 eV; and acquisition mass range, 50–550 m/z. The essential oil components were identified by comparing their calculated Kovats index (KI) with those available in the NIST and Wily databases.

GC-FID: The GC analysis was performed with an Agilent 7890B coupled with a flame ionization detector (FID). The column specifications as well as the thermal program of the column were similar to GC-MS. The injector and detector temperatures were set at 280 and 290 °C, respectively. Similar to GC-MS, helium with a flow rate of 1 mL/min was used as a carrier gas. The quantity of essential oil components was calculated from the GC peak areas using the normalization method.

**Table 1:** Geographical, climatic and soil characteristics of the studied *M. longifolia* accessions

Accessions	Country	Longitude (E)	Latitude (*N*)	Altitude (m)	Mean monthly temperature (°C)	Mean monthly precipitation (mm)	pH	E.C. (ds/m)	Clay (%)	Silt (%)	Sand (%)	Soil texture	Organic matter (%)	Nitrogen (%)	Phosphorus (ppm)	Potassium (ppm)
Isiveh	Iraq	45°16’58.4”	36°03’59.5”	864	20.9	38.04	7.67	0.554	31.76	23.84	44.4	Clay loam	1.42	0.14	11.2	313
Soni	Iraq	45°14’05.7”	36°17’26.0”	988	20.9	38.04	7.41	0.544	9.76	17.84	72.14	Sandy loam	2	0.2	19	202
Sarouchavah	Iraq	44°45’18.6”	36°16’11.5”	572	21.8	33.32	7.7	0.583	55.76	27.84	16.4	Clay	1.64	0.16	1	374
Shaghlavah	Iraq	44°28’58.1”	36°17’22.3”	913	20.85	37.4	7.61	0.581	15.76	23.84	60.4	Sandy loam	2.16	0.22	59	273
Khabat	Iraq	43°39’01.1”	36°15’28.8”	245	23.69	25.9	7.62	0.573	25.76	41.84	32.4	Loam	2.15	0.2	6.3	18
Harir	Iraq	44°11’17.9”	36°37’38.4”	391	20.85	37.4	7.8	1.19	45.76	29.84	24.4	Clay	1.56	0.16	0	212
Baziyan	Iraq	45°11’25.4”	35°32’44.4”	767	18.14	54.46	7.97	0.595	41.76	33.84	24.4	Clay	1.07	0.11	10	202
Darbandikhan	Iraq	45°41’33.9”	35°04’53.9”	370	22.42	37.33	7.69	0.417	5.76	11.84	82.4	Loamy sand	0.39	0.04	53	20
Kounamasi	Iraq	45°26’18.5”	35°48’02.9”	855	13.34	61.12	7.6	0.655	19.76	37.84	42.4	Loam	1.5	0.15	5	101
Taghtagh	Iraq	44°35’38.9”	35°53’44.8”	339	24.47	19.95	7.6	0.79	15.76	33.74	50.4	Loam	0.68	0.07	31	212
Marivan	Iran	46°12’18.1”	35°27’59.8”	1290	14.82	31.59	7.6	0.459	15.04	17.84	67.12	Sandy loam	0.55	0.05	17	81
Sanandaj	Iran	47°00’56.7”	35°16’20.9”	1320	16.71	17.73	7.7	0.626	25.4	35.84	39.12	Loam	1.81	0.18	49	536
Broujerd	Iran	48°40’33.7”	33°56’58.3”	1682	17.5	28.6	7.34	0.685	17.04	39.84	43.12	Loam	2.9	0.3	77	971
Khorramabad	Iran	48°17’30.5”	33°37’41.0”	1326	19.53	25.58	7.9	0.86	43.04	31.84	25.12	Clay	0.59	0.06	18	374
Kermanshah	Iran	47°01’19.1”	34°25’58.3”	1309	18.73	17.52	7.7	1.8	36.32	36.56	27.12	Clay loam	0.94	0.09	48	404
Kamyaran	Iran	46°53’33.7”	34°43’33.1”	1358	19.88	22.15	7.7	0.982	45.04	37.84	17.12	Clay	1.62	0.16	31	536
Divandarreh	Iran	46°54’44.6”	36°03’41.3”	2137	10.97	18.92	7.9	0.63	29.04	29.84	41.12	Clay loam	1.87	0.19	12	273
Saqez	Iran	46°17’29.2”	36°15’38.3”	1466	13.7	28.38	7.8	1	28.32	36.56	35.12	Clay loam	0.94	0.09	17.4	263
Boukan	Iran	46°06’36.1”	36°34’01.0”	1468	16.54	14.68	7.6	1.6	38.32	30.56	31.12	Clay loam	0.86	0.09	1.5	192
Mahabad	Iran	45°47’54.9”	36°49’28.9”	1460	15.49	25.01	7.8	0.791	36.32	38.56	25.12	Clay loam	1.85	0.18	18	435

### Antifungal activity

#### Fungal strain

**Fig. 2:**
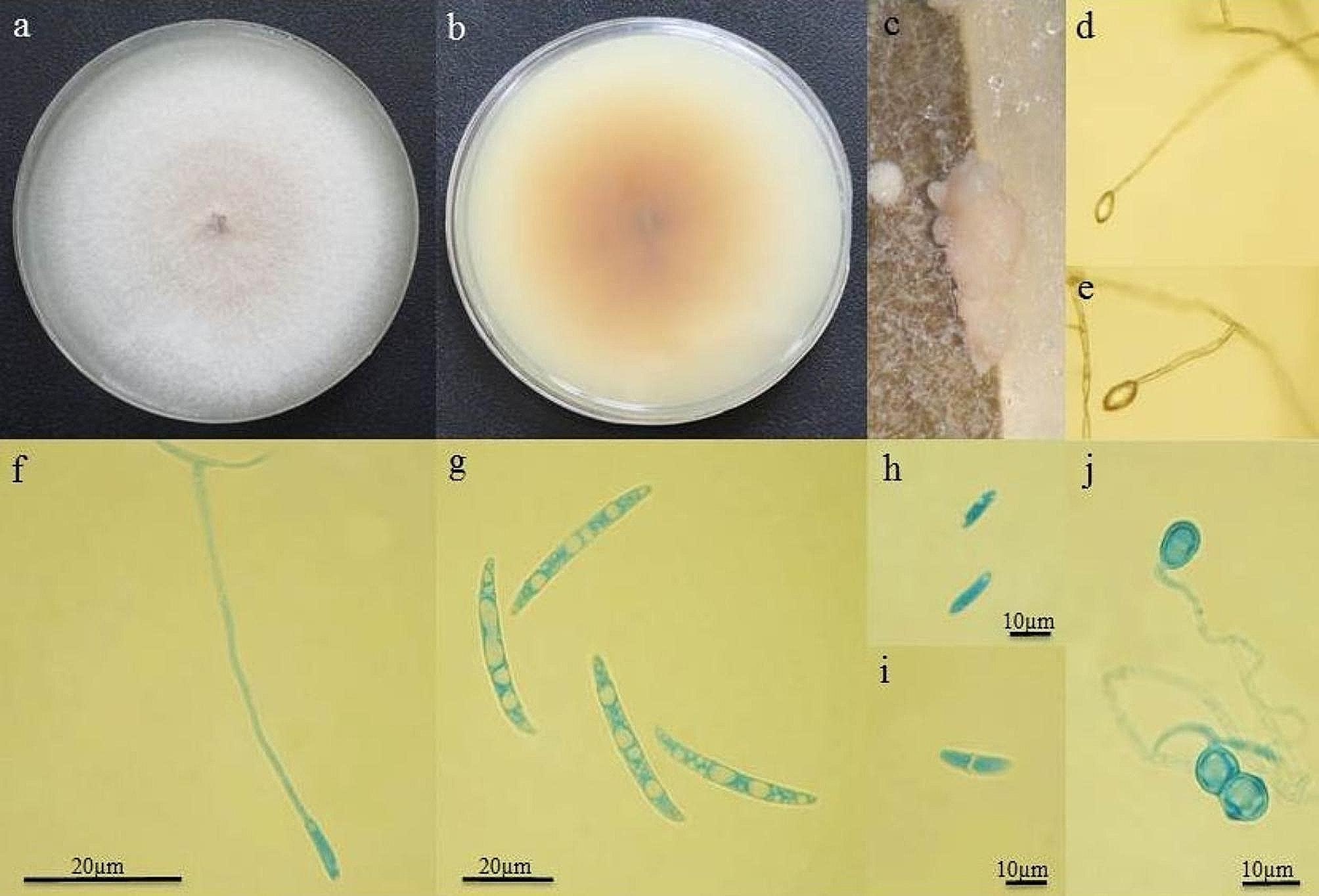
Morphology of *F. solani* fungus. **a** The upper surface of the fungus in the food medium of potato dextrose agar. **b** the bottom surface of the fungus in the food medium of potato dextrose agar. **c** Sporodocium on the leaf of clove plant. **d** and **e** false head. **f** conidial cell (monophyalid). **g** macroconidium. **h** and **i** microconidium. **j** chlamydospore

*Fusarium solani* (GenBank Acc. No. ON623894) used in the present study was obtained from the University of Kurdistan (Iran) (Fig. 2). Pure culture was transferred into petri dishes (diameter 90 mm) with potato dextrose agar (PDA) and incubated at 24 ± 2 °C for seven days and stored at 4 °C for long-term use.

### Determination of the fumigation and contact activities of essential oils on mycelia growth

The antifungal activity of essential oils against *F. solani* was investigated in laboratory conditions using contact and fumigation methods in PDA. In the contact method, different concentrations of essential oils (100, 250, 500, 750 and 1000 ppm) were dissolved in PDA medium containing Tween 80 (0.5% v/v). 15 ml of the obtained solutions were pipetted into plates (9 cm diameter), and the plates were allowed to solidify. A five-day-old disc plug mycelium (5 mm diameter) of *F. solani* was placed in the center of each plate. PDA without essential oil was used as a control. The plates were incubated at a temperature of 24 ± 2 °C. After ten days, the diameter of *F. solani* colonies was measured, and the inhibition percentage (I) was calculated using the following formula:

Inhibition (%) = [(dc -dt)]/dc×100.

In the above formula, dc is the radial growth of the pathogen in control; dt is the radial growth of the pathogen in the treatments. In the fumigation method, sterile filter paper discs (diameter 5 mm, Whatman No. 1) were placed for 60 s in different concentrations of essential oils and in the inverted lid of the plates. A mycelium disc plug of the pathogen (5 mm diameter) was taken from the periphery of a 5-day-old culture and placed in the center of the PDA culture medium. Then, the plates were tightly sealed with parafilm to prevent loss of volatile compounds. Plates containing filter paper discs immersed in sterile water were used as controls [36]. Each treatment consisted of four replications in both fumigation and contact methods. All the plates were incubated at 24 ± 2 °C and in dark condition. After 10 days, the diameter of *F. solani* colonies was measured in different plates, and the percentage of growth inhibition was evaluated. All experiments were performed twice with four replications.

### Morphological changes of mycelia in the presence of essential oil

Mycelia of *F. solani* were treated with *M. longifolia* essential oil at a concentration of 1000 ppm, based on contact and fumigation methods for seven days. Then, the mycelia were freeze-dried. Morphological changes of mycelia were observed using a scanning electron microscope (SEM) (Zeiss, Germany) at Kermanshah University of Medical Sciences, Kermanshah, Iran.

### Statistical analysis

The mean comparison of data (based on Duncan test at probability level 5%) was done using SPSS (ver. 21), two-way cluster analysis of accessions based on essential oil components was carried out using PAST (ver. 4), and heat map correlation between essential oil content and components with climatic and edaphic characteristics, and also essential oil components with inhibition percentage of mycelia growth was obtained using GraphPad Prism (ver. 8).

## Results and discussion

### Essential oil content and components

**Fig. 3:**
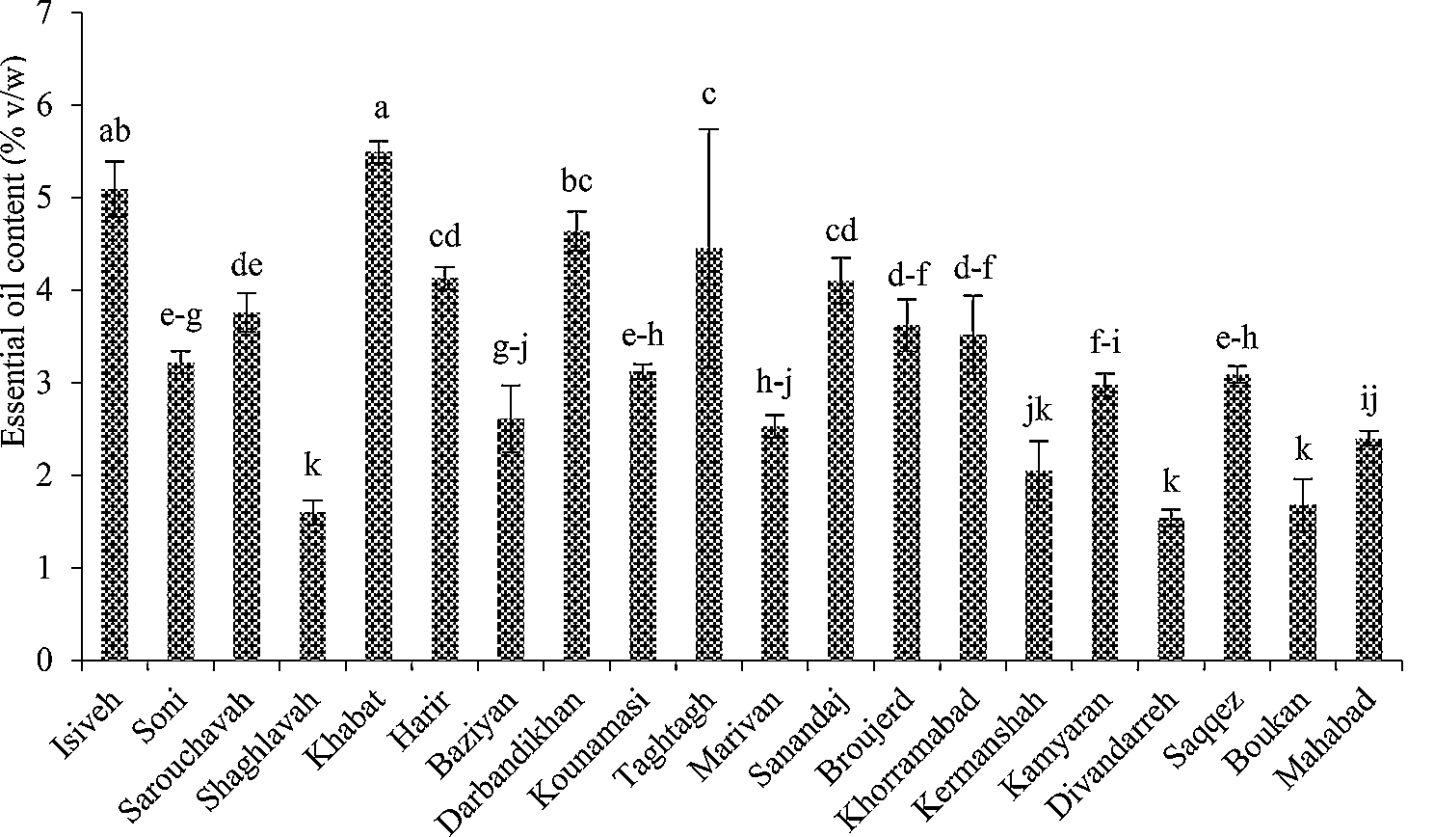
Essential oil content of the studied *M. longifolia* accessions

A considerable diversity was observed in the viewpoint of essential oil content among the studied *M. longifolia* accessions. The highest essential oil content (5.49 ± 0.12%) belonged to Khabat accession, followed by Isiveh (5.09 ± 0.3%), and the lowest essential oil content (1.54 ± 0.09%) was obtained from the Divandarreh accession, however, it was not significantly different from the Shaghlavah (1.6 ± 0.13%), Boukan (1.68 ± 0.28%), and Kermanshah (2.05 ± 0.32%) (Fig. 3). In total, 27 compounds were identified in the essential oil of the studied accessions, which accounted for 85.5-99.61% of the essential oil components, depending on the accession. The number of identified compounds was different in the studied accessions. The highest number of identified compounds (25) belonged to Shaghlavah and the lowest of them was obtained for Divandarreh and Taghtagh accessions (17). In all accessions, oxygenated monoterpenes were the dominant class of compounds in the essential oil, and the highest (94.08%) and lowest (58.15%) of these compounds were found in Broujerd and Shaghlavah accessions, respectively. The highest amount of monoterpene hydrocarbons (32.06%), sesquiterpene hydrocarbons (9.2%), and oxygenated sesquiterpenes (0.41%) were obtained from the essential oil of Shaghlavah, Shaghlavah, and Soni accessions, respectively. Oxygenated sesquiterpenes were not found in the essential oil of Harir, Darbandikhan, Taghtagh, Khorramabad, Divandarreh, and Mahabad accessions. The dominant constituents in the essential oils were different, depending on the accession. 1,8-cineole and isopulegyl acetate in Isiveh, Harir, and Darbandikhan; pulegone and isopulegyl acetate in Soni and Mahabad; 1,8-cineole, pulegone, and isopulegyl acetate in Sarouchavah and Khorramabad; pulegone and 1,8-cineole in Kounamasi and Boukan; neo-dihydro carveol acetate, limonene, and *α*-terpineol in Shaghlavah; pulegone and menthone in Khabat; 1,8-cineole, pulegone, and menthone in Baziyan; pulegone, 1,8-cineole, isopulegyl acetate, and menthone in Taghtagh; pulegone, piperitenone oxide, isopulegyl acetate, and piperitenone in Marivan; piperitenone and piperitenone oxide in Sanandaj; piperitenone oxide, pulegone, and piperitenone in Broujerd; bornyl acetate, piperitenone oxide, and *p*-menth-3-en-8-ol in Kermanshah; pulegone and piperitenone in Kamyaran; isopulegyl acetate, pulegone, piperitenone oxide, and bornyl acetate in Divandarreh; and pulegone, limonene, and isopulegyl acetate in Saqqez, were the dominant constituents of the essential oil (Table 2).

The essential oil content of *M. longifolia* in previous studies has been obtained between 1 and 2% [1], 0.87–1.83% [16], 1-2.25% [17], 0.38–4.33% [18], 0.86–2.46% [19], 0.59–1.46% [20], and 1.17–5.5% [21].

Similar to our results, in previous studies, oxygenated monoterpenes have been identified as the main class of compounds in the *M. longifolia* essential oil, and their amount has varied between 52.28 and 52.7% [18], 52.4-52.85% [1], 56.5-56.89% [20], and 85.76–92.03% [21]. In previous reports, thymol, carvacrol, carvacrol acetate and *p*-cymene [1], *β*-ocimene [18], 1,8-cineole [1, 18, 21, 23], menthone [18, 21, 22], menthol [18, 19], pulegone [18, 21–23], piperitenone [18, 23], piperitenone oxide [18–23], piperitone oxide [18–20, 22], *trans*-dihydrocarvone [19], menthofuran [20], and citronellyl acetate and aromandrene [23] have been reported as the dominant components of *M. longifolia* essential oil. The variation in the essential oil content and composition of different accessions belonging to a specific plant species can be due to their genetic background or habitat conditions [37]. The production of secondary metabolites in plants is influenced by two factors: the genetic potential of the plant (constitutive production) and external biotic and abiotic stresses (induced production) [38]. Based on the above references, the observed diversity in the phytochemical profile of the studied *M. longifolia* accessions might be due to the difference in their genetic properties or habitat conditions.

**Table 2:** Chemical composition of essential oil in the studied *M. longifolia* accessions

Compound	KIc	KIr	Isiveh	Soni	Sarouchavah	Shaghlavah	Khabat	Harir	Baziyan	Darbandikhan	Kounamasi	Taghtagh
*α*-Pinene	929	932	2.05	0.61	2.35	2.68	1.30	2.70	1.24	2.98	1.38	1.44
Sabinene	968	969	-	0.46	2.57	2.41	1.26	4.11	1.93	-	1.15	2.37
*β*-Pinene	971	974	0.88	0.80	2.61	3.93	1.96	1.18	2.88	-	2.36	1.86
*β*-Myrcene	989	988	3.83	0.54	1.68	2.78	1.15	0.98	2.31	6.94	1.47	0.50
*α*-Terpinene	1012	1014	1.54	-	-	-	-	-	-	3.53	-	-
*p*-Cymene	1020	1024	-	1.02	-	-	-	-	-	-	-	-
Limonene	1024	1025	0.09	1.09	-	11.30	4.16	-	-	-	-	-
1,8-Cineole	1027	1026	39.22	0.91	37.06	9.62	8.44	43.52	38.75	62.18	18.50	26.96
*γ*-Terpinene	1056	1059	-	0.05	0.08	0.60	-	0.20	-	0.02	-	-
*cis*-Sabinene	1061	1065	0.17	0.40	0.07	7.61	0.12	0.38	0.24	0.35	0.13	0.10
*α*-Terpinolene	1085	1086	0.33	-	-	0.21	0.07	0.10	-	-	-	-
*trans*-Sabinene hydrate	1094	1098	0.06	-	0.02	0.54	-	0.21	0.09	-	-	0.09
*p*-Menth-2-en-1-ol	1114	1118	0.06	0.04	0.08	0.49	-	0.12	0.11	0.08	0.05	0.07
*p*-Menth-3-en-8-ol	1143	1145	-	0.03	2.28	0.29	-	0.03	0.06	0.11	-	-
Menthone	1151	1148	1.83	0.14	2.86	0.44	25.18	7.83	16.97	0.76	7.14	14.04
Isopulegol	1170	1169	0.07	0.05	0.07	0.48	0.03	0.18	0.17	0.08	0.03	0.14
*α*-Terpineol	1184	1186	1.11	0.06	2.56	11.19	1.11	2.44	2.68	0.25	1.87	2.84
Pulegone	1235	1233	3.41	56.03	27.31	5.97	52.60	5.20	26.25	7.73	62.09	30.22
Isopulegyl acetate	1274	1275	42.02	27.72	14.82	0.56	0.27	24.94	0.34	11.33	0.28	16.76
Bornyl acetate	1286	1284	0.09	-	0.32	0.31	0.07	0.77	0.06	0.16	0.08	0.04
Neo-Dihydro carveol acetate	1303	1306	0.08	-	0.05	20.76	0.04	0.36	0.06	0.04	0.04	-
Piperitenone	1341	1340	0.17	6.71	0.27	7.91	0.55	0.31	0.17	0.20	0.83	0.31
Piperitenone oxide	1368	1366	0.28	0.06	-	0.13	-	0.92	0.02	0.35	-	-
*trans*-Caryophyllene	1411	1417	0.78	1.21	1.84	6.68	0.79	0.39	1.71	0.86	1.07	0.95
Germacrene D	1489	1484	0.25	0.38	0.23	2.09	-	0.11	0.24	0.12	0.31	0.12
Bicyclogermacrene	1497	1500	0.12	0.12	0.06	0.43	0.02	-	0.10	0.15	0.10	-
Caryophyllene oxide	1581	1582	0.08	0.41	0.17	0.20	0.03	-	0.10	-	0.08	-
Monoterpene hydrocarbons			8.95	4.97	9.38	32.06	10.02	9.86	8.69	13.82	6.49	6.36
Oxygenated monoterpenes			88.34	91.75	87.68	58.15	88.29	86.62	85.64	83.27	90.91	91.38
Sesquiterpene hydrocarbons			1.15	1.71	2.13	9.20	0.81	0.50	2.05	1.13	1.48	1.07
Oxygenated sesquiterpenes			0.08	0.41	0.17	0.20	0.03	-	0.10	-	0.08	-
Total identified compounds			98.52	98.84	99.36	99.61	99.15	96.98	96.48	98.22	98.96	98.81
**Compound**	**KIc**	**KIr**	**Marivan**	**Sanandaj**	**Broujerd**	**Khorramabad**	**Kermanshah**	**Kamyaran**	**Divandarreh**	**Saqqez**	**Boukan**	**Mahabad**
*α*-Pinene	929	932	0.78	0.92	0.56	1.48	2.10	1.20	0.27	1.71	0.61	0.70
Sabinene	968	969	0.48	0.56	0.86	0.65	1.34	0.40	1.57	1.54	0.89	0.37
*β*-Pinene	971	974	0.81	1.01	0.33	1.64	2.31	1.43	1.21	3.07	0.47	0.72
*β*-Myrcene	989	988	0.46	0.70	-	2.19	1.70	1.30	-	1.93	-	0.31
*α*-Terpinene	1012	1014	0.08	0.09	-	0.60	0.68	-	-	-	0.22	0.37
*p*-Cymene	1020	1024	2.44	-	-	-	-	-	-	-	-	0.15
Limonene	1024	1025	3.47	1.29	1.01	8.59	2.92	6.78	5.67	19.07	-	1.26
1,8-Cineole	1027	1026	1.13	5.99	6.17	16.19	4.96	2.33	4.66	9.66	27.56	1.31
*γ*-Terpinene	1056	1059	0.03	1.93	-	-	-	-	-	0.63	0.04	0.02
*cis*-Sabinene	1061	1065	0.25	-	0.10	0.05	0.19	0.17	-	0.04	-	0.03
*α*-Terpinolene	1085	1086	-	-	-	-	0.03	0.02	-	0.02	-	-
*trans*-Sabinene hydrate	1094	1098	-	0.03	0.15	0.09	0.04	-	-	0.03	0.10	-
*p*-Menth-2-en-1-ol	1114	1118	0.11	0.03	0.16	0.11	0.07	0.13	0.30	-	0.09	0.08
*p*-Menth-3-en-8-ol	1143	1145	0.46	0.03	0.13	0.05	11.98	3.10	0.11	-	0.71	0.14
Menthone	1151	1148	0.21	0.17	-	6.02	0.91	5.34	3.54	9.15	-	4.06
Isopulegol	1170	1169	-	0.02	0.06	0.13	-	-	-	0.05	0.06	0.42
*α*-Terpineol	1184	1186	0.04	0.22	0.29	1.37	1.31	0.32	3.57	2.18	1.27	1.66
Pulegone	1235	1233	30.12	1.31	25.20	26.04	5.72	24.39	19.46	30.10	34.11	57.28
Isopulegyl acetate	1274	1275	16.09	0.69	8.92	17.01	5.06	9.30	21.14	15.83	9.22	27.66
Bornyl acetate	1286	1284	0.27	0.40	-	0.55	33.24	0.29	14.15	0.53	0.10	0.12
Neo-Dihydro carveol acetate	1303	1306	-	-	-	0.03	-	0.21	-	0.04	-	0.08
Piperitenone	1341	1340	14.16	51.94	17.13	9.42	-	23.99	1.49	0.30	0.11	0.08
Piperitenone oxide	1368	1366	17.99	29.09	36.02	5.71	19.05	2.66	17.45	0.20	9.24	0.10
*trans*-Caryophyllene	1411	1417	1.39	2.18	0.72	0.70	3.54	1.27	2.51	1.48	2.75	0.70
Germacrene D	1489	1484	0.50	0.49	0.57	0.21	1.20	0.52	1.12	0.54	1.01	0.38
Bicyclogermacrene	1497	1500	0.11	0.19	0.14	0.12	0.20	0.32	0.24	-	0.29	0.39
Caryophyllene oxide	1581	1582	0.17	0.31	0.07	-	0.14	0.03	-	0.08	0.29	-
Monoterpene hydrocarbons			8.80	6.53	3.01	15.29	11.31	11.30	8.72	28.04	2.33	3.93
Oxygenated monoterpenes			80.58	89.89	94.08	82.63	82.30	72.06	85.87	68.04	82.47	93.0
Sesquiterpene hydrocarbons			2.00	2.86	1.43	1.03	4.94	2.11	3.87	2.02	4.05	1.47
Oxygenated sesquiterpenes			0.17	0.31	0.07	-	0.14	0.03	-	0.08	0.29	-
Total identified compounds			91.55	99.59	98.59	98.95	98.69	85.50	98.46	98.18	89.14	98.39

KIc: calculated Kovats retention index, KIr: references Kovats retention index

**Fig. 4:**
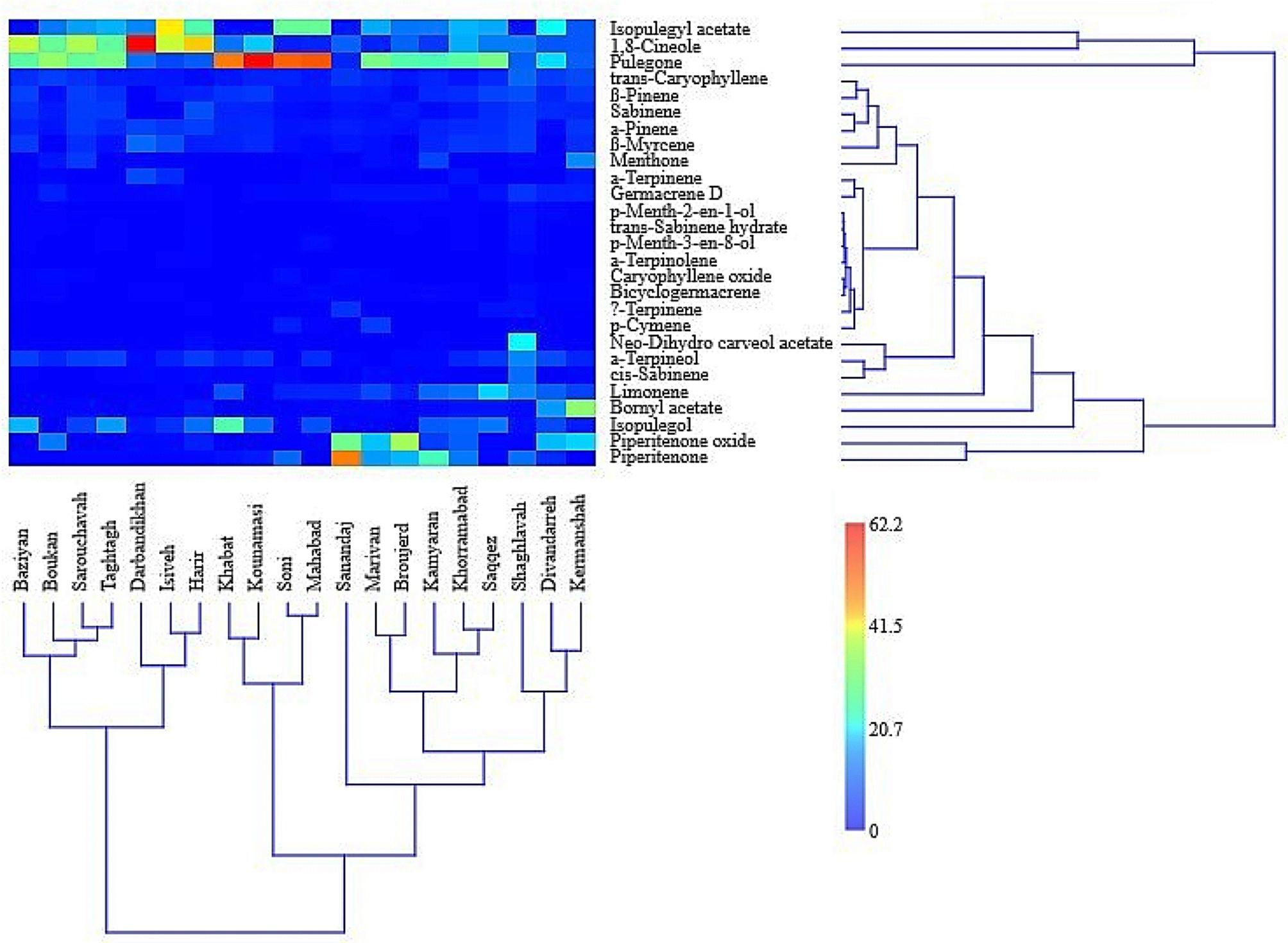
Two-way cluster analysis of *M. longifolia* accessions based on their essential oil components

Cluster analysis of *M. longifolia* accessions based on total essential oil compounds grouped them into three clusters. The first cluster included Baziyan, Boukan, Sarouchavah, Taghtagh, Darbandikhan, Isiveh and Harir. The second cluster included Khabat, Kounamasi, Soni and Mahabad, and other accessions were included in the third cluster. The grouping of accessions in different clusters was not in accordance with their geographical distance, and this result indicates the greater importance of genetic factors compared to habitat conditions in the separation of accessions (Fig. 4).

### Correlation of essential oil content and components with climatic and edaphic characteristics

**Fig. 5:**
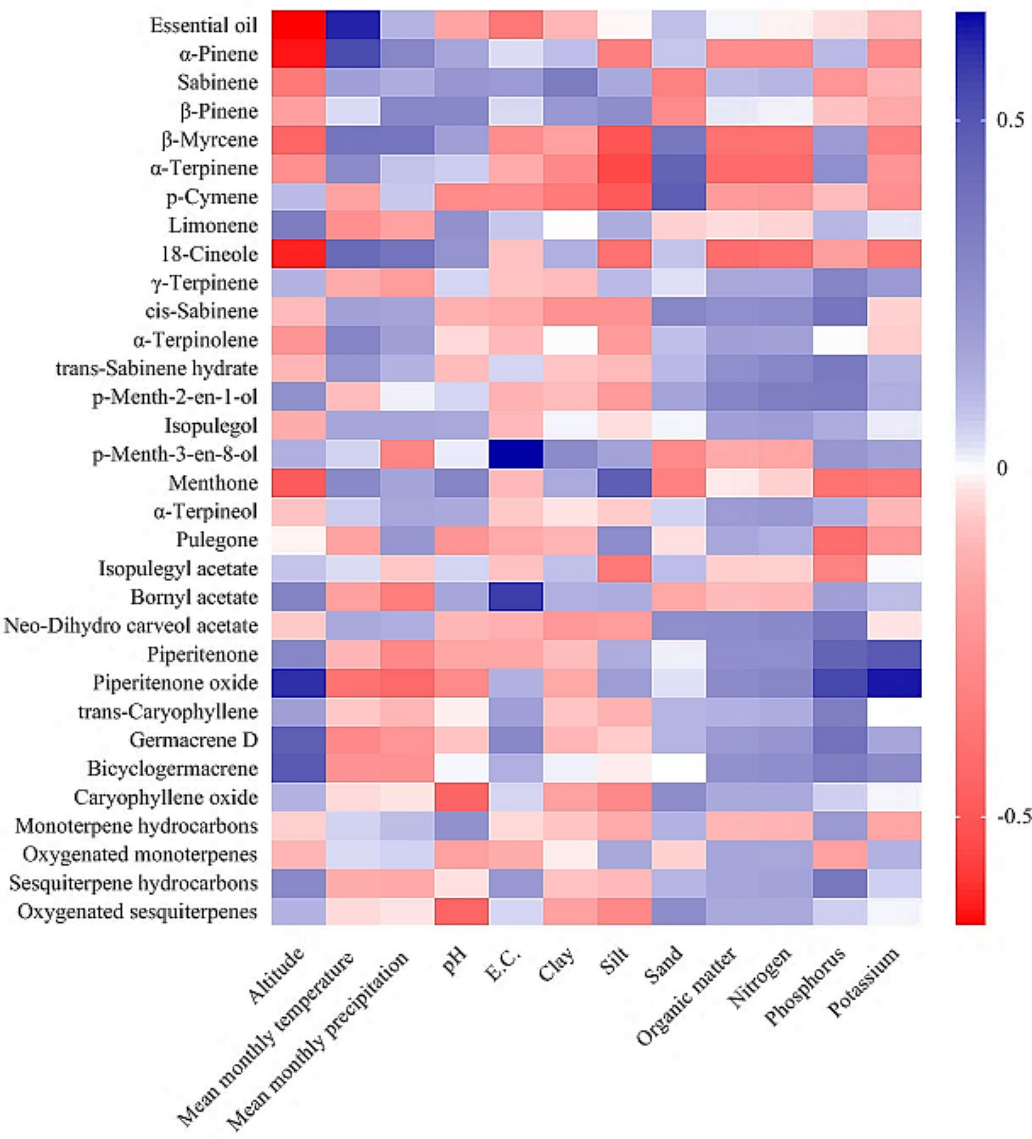
Heat map correlation between essential oil content and components of the studied *M. longifolia* accessions with climatic and edaphic characteristics of their habitats

The essential oil content showed a significant positive correlation with the mean monthly temperature (*r* = 0.617) and a significant negative correlation with the altitude of the habitat (*r*= -0.657). Essential oil content had no significant correlation with soil characteristics. Among the essential oil components, *α*-pinene, *β*-myrcene, 1,8-cineole, and menthone had significant negative correlations with altitude (*r*= -0.638, *r*= -0.447, *r*= -0.622, and *r*= -0.487, respectively), while piperitenone oxide, germacrene D, and bicyclogermacrene showed significant positive correlations with altitude (*r* = 0.598, *r* = 0.471, and *r* = 0.487, respectively). Also, *α*-pinene showed a notable positive correlation with the mean monthly temperature of the habitat (*r* = 0.535) (Fig. 5).

The other significant correlations were included the positive correlations of *p*-menth-2-en-1-ol and bornyl acetate with electrical conductivity (*r* = 0.657 and *r* = 0.571, respectively), menthone with silt (*r* = 0.472), *α*-terpinene and *p*-cymene with sand (*r* = 0.453 and *r* = 0.476, respectively), piperitenone and piperitenone oxide with phosphorus (*r* = 0.450 and *r* = 0.544, respectively) and potassium (*r* = 0.492 and *r* = 0.630, respectively), and also the negative correlations of caryophyllene oxide with pH (*r*= -0.446), *β*-myrcene, *α*-terpinene, and *p*-cymene with silt (*r*= -0.510, *r*= -0.548, and *r*= -0.488, respectively), and oxygenated sesquiterpenes with pH (*r*= -0.446) (Fig. 5).

The optimum temperature for essential oil production in *M. longifolia* has been reported between 18 and 20 °C [39]. The increase in essential oil production at relatively high temperatures may be due to the increase in photosynthesis and the activity of enzymes responsible for essential oil biosynthesis [40]. Essential oils have a high heat capacity and can prevent damage to the plant in heat stress conditions by storing heat [41]. Negative correlations between altitude and essential oil content have been reported in previous studies conducted on *M. longifolia* [16, 18, 19], *Thymus migricus* [42], *Thymus kotschyanus* [43], and *Achillea millefolium* L. subsp. *millefolium* [44]. It seems that the decrease in essential oil production at higher altitudes is due to the decrease in temperature. Low temperatures cause a decrease in nutrient uptake, photosynthesis and carbohydrate production, and subsequently lead to a decrease in secondary metabolites production [45, 46].

The correlation of *M. longifolia* essential oil components with habitat conditions has been less studied. Afkar et al. [23]. found significant positive correlations between pulegone with annual precipitation and mean annual temperature; piperitenone with soil pH, *α*-pinene with soil sand content; and significant negative correlations between1,8-cineole with altitude; menthone and *α*-terpineol with soil clay content.

### Antifungal activity

#### Scanning electron microscopy analysis

**Fig. 6:**
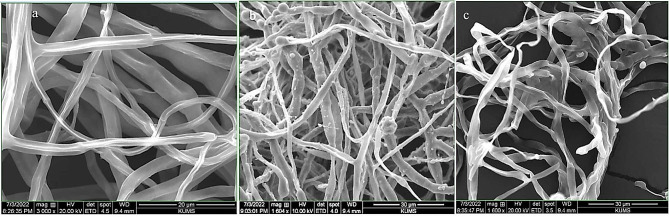
Morphological changes of *F. solani* hyphae. **a** Control sample. **b** Treated with essential oil via contact method. **c** Treated with essential oil via fumigation method

The mycelia morphology of *F. solani* treated with *M. longifolia* essential oil at a concentration of 1000 µl/l was observed by SEM. In the control sample, the mycelia were regular, uniform and complete with smooth surfaces, but the mycelia in the samples treated with essential oil showed many morphological changes, including irregular growth, formation of verrucous surface, shrinkage, collapse and hollowing of hyphae (Fig. 6).

#### Inhibition activity of essential oils on mycelia growth

The results indicated that *M. longifolia* essential oil inhibited the growth of the fungus mycelia in both contact and fumigation methods (Table 3). In all accessions, the fumigation method compared to the contact method was more able to control mycelia growth. The effect of essential oils on fungus growth is strongly influenced by the method used [47]. Different studies have reported that fumigation method is more effective in controlling the fungus than the contact method [48–50]. Monoterpenes are the main class of compounds in *M. longifolia* essential oil, which have high volatility, and in the fumigation method, they easily convert from liquid to vapor phase, absorbed by the fungus mycelia and finally prevent its growth. But in the contact method, since the essential oil is dissolved in the liquid phase, monoterpenes vaporization and absorption by the fungus mycelia are difficult, and as a result, the fungus continues to grow [51]. In both methods, the inhibition percentage of essential oil on mycelia growth increased with an increase in essential oil concentration. The essential oil concentration is one of the important factors in its effectiveness [52].

The highest inhibition percentage in the fumigation method (100%) belonged to the essential oil of Mahabad, Divandarreh and Kermanshah accessions, while the lowest was seen for Taghtagh, Boukan and Harir (45.28, 48.34 and 48.61%, respectively). In the contact method, the highest inhibition percentage was observed in Broujerd, Kermanshah and Saqez accessions (77.78, 75.84 and 74.17%, respectively), and the lowest was for Darbandikhan and Divandarreh accessions (11.11 and 13.89%, respectively) (Table 3).

**Table 3:** Inhibition activity (%) of essential oils of *M. longifolia* accessions on mycelia growth of *F. solani* after 10 days using two contact and fumigation methods

Accession	Concentrations									
	100 ppm		250 ppm		500 ppm		750 ppm		1000 ppm	
	Contact	Fumigation	Contact	Fumigation	Contact	Fumigation	Contact	Fumigation	Contact	Fumigation
Isiveh	4.45f	21.39e-g	14.16d-g	25.00de	17.78i-l	31.11 h	21.94jk	43.61f	29.72i	56.11 g
Soni	7.78ef	15.84 h-j	11.39e-g	28.89d	20.00 g-j	38.89 g	26.67 g-i	50.28e	32.22hi	74.72de
Sarouchavah	20.28b	26.39d	28.06b	37.23c	33.33b	43.61f	41.67d	52.50de	53.34d	63.89f
Shaghlavah	6.39ef	10.00k	11.11 fg	26.11de	16.95kl	53.06d	21.11jk	63.33c	42.78e	78.89c
Khabat	9.72de	13.89j	20.83 cd	29.17d	26.11 cd	51.39de	31.67f	59.73c	36.11f-h	72.22e
Harir	16.39bc	20.00 fg	18.06c-f	28.89d	23.89de	32.22 h	29.45 fg	40.56 fg	32.50hi	48.61 h
Baziyan	5.56ef	14.45j	16.11c-g	20.83f	20.28 g-i	25.28i	23.61i-k	31.67 h	38.61 fg	58.06 g
Darbandikhan	0.00 g	34.17c	2.50 h	40.83c	4.72 m	44.16f	7.78 L	54.72d	11.11j	66.39f
Kounamasi	7.23ef	15.00ij	10.28 g	26.11de	15.84 L	41.67 fg	20.56k	53.61de	66.67c	79.45c
Taghtagh	5.56ef	21.39e-g	15.00d-g	26.11de	19.17 h-k	34.72 h	23.06i-k	39.45 g	34.45gh	45.28 h
Marivan	8.06ef	17.23 g-j	11.67e-g	26.67de	20.56f-h	33.05 h	27.78f-h	40.00 g	43.05e	63.33f
Sanandaj	16.67bc	18.89f-i	18.34c-e	24.17ef	23.06ef	31.39 h	30.28 fg	39.72 g	36.39f-h	54.72 g
Broujerd	35.28a	38.89b	41.67a	60.83a	47.78a	77.78b	69.17a	84.17b	77.78a	93.06b
Khorramabad	14.45c	21.39e-g	28.06b	27.22de	21.94e-g	39.45 g	29.72 fg	53.33de	38.89f	73.61e
Kermanshah	7.23ef	21.11e-g	12.78e-g	47.22b	17.50j-l	82.50a	49.45c	100.00a	75.84a	100.00a
Kamyaran	17.50bc	24.45de	21.94bc	37.78c	26.11 cd	48.89e	31.39f	60.56c	43.33e	77.23 cd
Divandarreh	0.00 g	36.11bc	2.50 h	48.61b	4.16 m	73.89c	6.95 L	100.00a	13.89j	100.00a
Saqez	13.89 cd	19.17f-h	22.22bc	27.50de	35.56b	53.89d	56.11b	62.22c	74.17ab	80.00c
Boukan	9.17e	22.50ef	15.84c-g	27.50de	20.56f-h	33.06 h	24.72 h-j	39.17 g	34.73f-h	48.34 h
Mahabad	9.72de	45.55a	18.34c-e	60.56a	27.78c	75.00bc	37.50e	87.23b	70.83b	100.00a

In each column, the means with the same letters do not have a significant difference at the 5% probability level

**Fig. 7:**
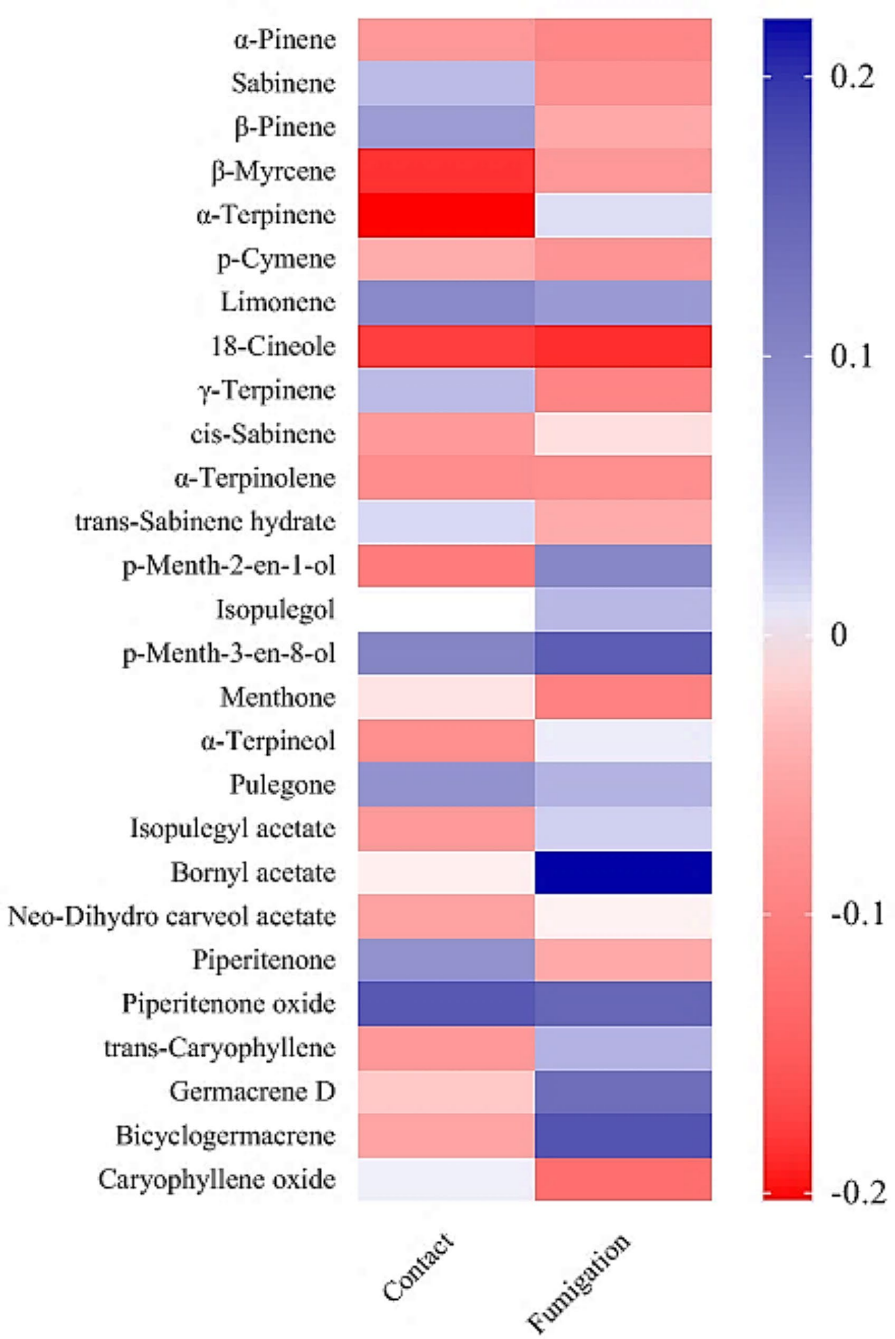
Heat map correlation between the inhibition percentage of mycelium growth with essential oil components

The essential oils of the studied accessions were different in terms of the number, type and amount of their components, which can affect their fungicidal potential. Significant correlations were found between the essential oil components and the inhibition percentage of mycelium growth. In the contact method, a notable negative correlation was obtained between the inhibition percentage with *β*-myrcene (*r*= -0.184), *α*-terpinene (*r*= -0.203), 1,8-cineole (*r*= -0.176) and p-menth-2-en-1-ol (*r*= -0.106). In contrast, the inhibition percentage showed a significant positive correlation with limonene (*r* = 0.096), p-menth-3-en-8-ol (*r* = 0.102) and piperitenone oxide (*r* = 0.167). In the fumigation method, a significant negative correlation was observed between the inhibition percentage with *α*-pinene (*r*= -0.091), 1,8-cineole (*r*= -0.187), *γ*-terpinene (*r*= -0.094), menthone (*r*= -0.100) and caryophyllene oxide (*r*= -0.126). Also, a significant positive correlation was found between the inhibition percentage with *p*-menth-2-en-1-ol (*r* = 0.098), *p*-menth-3-en-8-ol (*r* = 0.161), bornyl acetate (*r* = 0.221), piperitenone oxide (*r* = 0.149), germacrene D (*r* = 0.137) and bicyclogermacrene (*r* = 0.172) (Fig. 7).

The effectiveness of essential oils strongly depends on their phytochemical profile [53]. It has been reported that there is a close relationship between the chemical structure of essential oil compounds and the fungicidal activity of essential oil [54]. The most antifungal activity of essential oil components is related to phenolic compounds, followed by aldehydes, ketones, alcohols, ethers, and hydrocarbons [52]. It has been reported that the antifungal activity of *M. longifolia* essential oil is closely related to the amount of oxygenated monoterpenes [55]. Among the essential oil components that showed significant correlations with the inhibition percentage of mycelium growth, *α*-pinene, *β*-myrcene, *α*-terpinene, limonene, *γ*-terpinene, germacrene D, and bicyclogermacrene are hydrocarbon compounds; 1,8-cineole and caryophyllene oxide belong to ethereal compounds; *p*-menth-2-en-1-ol and *p*-menth-3-en-8-ol are grouped in alcoholic compounds; menthone, bornyl acetate and piperitenone oxide are ketone compounds. It should be noted that the antifungal activity of essential oil is not always dependent on a specific component, and sometimes the synergistic effects of components determine the level of its antifungal activity [56]. Essential oils inhibit the growth of pathogenic fungi by inhibiting the function of fungal mitochondria, preventing cell wall formation, inhibiting efflux pumps, and destroying the cell membrane [57].

The antifungal activity of some essential oil components has been proven in previous studies [58–67], although the mechanism of their action is not well understood. Bornyl acetate impacts the growth of fungus by affecting enzyme activity, as well as penetrating the lipids of the mitochondrial and cytoplasmic membranes, and as a result, disrupting their structure [58]. Moreover, bornyl acetate affects gene expression and protein activity in different cell types [59]. The antifungal activity of *Mentha suaveolens* essential oil has been attributed to the high amount of piperitenone oxide [60]. An increase in permeability and damage to the cell membrane, the failure of ion transfer and ATP generation, the disruption of intracellular ion homeostasis, the leakage of intracellular proteins, and the change in cell morphology are some of the limonene effects on pathogenic fungi [61]. The fungicidal potential of germacrene and caryophyllene oxide has also been proven, but the mechanism of their action is not exactly known [62, 63]. In contrast to our results, *α*-pinene has been shown to have strong antifungal activity [64–67]. Alpha-pinene damages the cell membrane and affects its conductivity and ion leakage [64]. The antifungal activity of *α*-pinene may be related to ergosterol binding in the fungal cytoplasmic membrane [65]. It has been proven that *α*-pinene inhibits the growth of fungal structures such as pseudo-hyphae and blastoconidia [66]. The strong antifungal activity of *α*-pinene can be related to its inhibitory effect on the activity of phospholipase and esterase enzymes [67]. The inconsistency of our results with previous studies may be due to the synergistic effects of *α*-pinene and other essential oil compounds of *M. longifolia*, the type of application method, and the type of fungus studied. Essential oils are a mixture of several compounds, so to determine which component is responsible for the antifungal activity, it is necessary to purify the components and study their effects separately.

## Conclusions

The studied *M. longifolia* accessions showed significant differences in terms of the essential oil content and components. The dominant components of essential oil were different depending on the accessions. The phytochemical profile of accessions was more influenced by genetic than habitat conditions. Significant correlations were found between the essential oil content and components with climate and soil properties of habitat, which can be used for domestication and cultivation of this plant species. *M. longifolia* essential oil was able to prevent the growth of *F. solani* fungus, which indicates its strong antifungal activity. The fumigation method was found to be a better method to control the *F. solani* compared to the contact method. Significant correlations were observed between essential oil components and antifungal activity, which can be useful for producing biological toxins with the desired quality. Determining and introducing the elite accession can be different depending on the aim. If the aim is more essential oil content, Khabat and Isiveh were superior accessions, but if essential oil quality based on some specific compounds is the main goal, the superior accession can be different. The superior accession in the viewpoint of antifungal activity differed depending on the application method and concentration of the essential oil.

## Data Availability

The datasets used and/or analyzed during the current study are available from the corresponding author on reasonable request.
