# Correction: Phytochemical profile and antifungal activity of essential oils obtained from different *Mentha longifolia* L. accessions growing wild in Iran and Iraq

**DOI:** 10.1186/s12870-024-05237-8

**Published:** 2024-06-13

**Authors:** Kosrat Hama Mustafa, Jalal Khorshidi, Yavar Vafaee, Azad Rastegar, Mohammad Reza Morshedloo, Somaieh Hossaini

**Affiliations:** 1 Department of Horticultural Science and Engineering, Faculty of Agriculture, University of Kurdistan, Sanandaj, 66177-15175 Iran; 2 Department of Horticulture, College of Agricultural Engineering Sciences, University of Raparin, Ranya, Iraq; 3 Research Center of Medicinal Plants Breeding and Development, University of Kurdistan, Sanandaj, Iran; 4 Forests and Rangelands Research Department, Kurdistan Agricultural and Natural Resources Research and Education Center, Sanandaj, Iran; 5 Department of Horticultural Science and Engineering, Faculty of Agriculture, University of Maragheh, Maragheh, Iran; 6 Department of Plant Protection, Faculty of Agriculture, University of Kurdistan, Maragheh, 66177-15175 Iran

**Correction: BMC Plant Biol 24, 461 (2024)**

10.1186/s12870-024-05135-z

Following publication of the original article [1], the authors identified errors in Table 2. Unfortunately, Table 2 continued was missed out during production and was not processed by production team.

Below is the Table 2 continued which should have been captured:

The original article [1] has been corrected.

**Table 2:** (continued)

Compound	KIc	KIr	Marivan	Sanandaj	Broujerd	Khorramabad	Kermanshah	Kamyaran	Divandarreh	Saqqez	Boukan	Mahabad
*α*-Pinene	929	932	0.78	0.92	0.56	1.48	2.10	1.20	0.27	1.71	0.61	0.70
Sabinene	968	969	0.48	0.56	0.86	0.65	1.34	0.40	1.57	1.54	0.89	0.37
*β*-Pinene	971	974	0.81	1.01	0.33	1.64	2.31	1.43	1.21	3.07	0.47	0.72
*β*-Myrcene	989	988	0.46	0.70	-	2.19	1.70	1.30	-	1.93	-	0.31
*α*-Terpinene	1012	1014	0.08	0.09	-	0.60	0.68	-	-	-	0.22	0.37
*p*-Cymene	1020	1024	2.44	-	-	-	-	-	-	-	-	0.15
Limonene	1024	1025	3.47	1.29	1.01	8.59	2.92	6.78	5.67	19.07	-	1.26
1,8-Cineole	1027	1026	1.13	5.99	6.17	16.19	4.96	2.33	4.66	9.66	27.56	1.31
*γ*-Terpinene	1056	1059	0.03	1.93	-	-	-	-	-	0.63	0.04	0.02
*cis*-Sabinene	1061	1065	0.25	-	0.10	0.05	0.19	0.17	-	0.04	-	0.03
*α*-Terpinolene	1085	1086	-	-	-	-	0.03	0.02	-	0.02	-	-
*trans*-Sabinene hydrate	1094	1098	-	0.03	0.15	0.09	0.04	-	-	0.03	0.10	-
*p*-Menth-2-en-1-ol	1114	1118	0.11	0.03	0.16	0.11	0.07	0.13	0.30	-	0.09	0.08
*p*-Menth-3-en-8-ol	1143	1145	0.46	0.03	0.13	0.05	11.98	3.10	0.11	-	0.71	0.14
Menthone	1151	1148	0.21	0.17	-	6.02	0.91	5.34	3.54	9.15	-	4.06
Isopulegol	1170	1169	-	0.02	0.06	0.13	-	-	-	0.05	0.06	0.42
*α*-Terpineol	1184	1186	0.04	0.22	0.29	1.37	1.31	0.32	3.57	2.18	1.27	1.66
Pulegone	1235	1233	30.12	1.31	25.20	26.04	5.72	24.39	19.46	30.10	34.11	57.28
Isopulegyl acetate	1274	1275	16.09	0.69	8.92	17.01	5.06	9.30	21.14	15.83	9.22	27.66
Bornyl acetate	1286	1284	0.27	0.40	-	0.55	33.24	0.29	14.15	0.53	0.10	0.12
Neo-Dihydro carveol acetate	1303	1306	-	-	-	0.03	-	0.21	-	0.04	-	0.08
Piperitenone	1341	1340	14.16	51.94	17.13	9.42	-	23.99	1.49	0.30	0.11	0.08
Piperitenone oxide	1368	1366	17.99	29.09	36.02	5.71	19.05	2.66	17.45	0.20	9.24	0.10
*trans*-Caryophyllene	1411	1417	1.39	2.18	0.72	0.70	3.54	1.27	2.51	1.48	2.75	0.70
Germacrene D	1489	1484	0.50	0.49	0.57	0.21	1.20	0.52	1.12	0.54	1.01	0.38
Bicyclogermacrene	1497	1500	0.11	0.19	0.14	0.12	0.20	0.32	0.24	-	0.29	0.39
Caryophyllene oxide	1581	1582	0.17	0.31	0.07	-	0.14	0.03	-	0.08	0.29	-
Monoterpene hydrocarbons			8.80	6.53	3.01	15.29	11.31	11.30	8.72	28.04	2.33	3.93
Oxygenated monoterpenes			80.58	89.89	94.08	82.63	82.30	72.06	85.87	68.04	82.47	93.0
Sesquiterpene hydrocarbons			2.00	2.86	1.43	1.03	4.94	2.11	3.87	2.02	4.05	1.47
Oxygenated sesquiterpenes			0.17	0.31	0.07	-	0.14	0.03	-	0.08	0.29	-
Total identified compounds			91.55	99.59	98.59	98.95	98.69	85.50	98.46	98.18	89.14	98.39

**Continued Table 2.**

**Table:** 

Compound	KIc	KIr	Marivan	Sanandaj	Broujerd	Khorramabad	Kermanshah	Kamyaran	Divandarreh	Saqqez	Boukan	Mahabad
*α*-Pinene	929	932	0.78	0.92	0.56	1.48	2.10	1.20	0.27	1.71	0.61	0.70
Sabinene	968	969	0.48	0.56	0.86	0.65	1.34	0.40	1.57	1.54	0.89	0.37
*β*-Pinene	971	974	0.81	1.01	0.33	1.64	2.31	1.43	1.21	3.07	0.47	0.72
*β*-Myrcene	989	988	0.46	0.70	-	2.19	1.70	1.30	-	1.93	-	0.31
*α*-Terpinene	1012	1014	0.08	0.09	-	0.60	0.68	-	-	-	0.22	0.37
*p*-Cymene	1020	1024	2.44	-	-	-	-	-	-	-	-	0.15
Limonene	1024	1025	3.47	1.29	1.01	8.59	2.92	6.78	5.67	19.07	-	1.26
1,8-Cineole	1027	1026	1.13	5.99	6.17	16.19	4.96	2.33	4.66	9.66	27.56	1.31
*γ*-Terpinene	1056	1059	0.03	1.93	-	-	-	-	-	0.63	0.04	0.02
*cis*-Sabinene	1061	1065	0.25	-	0.10	0.05	0.19	0.17	-	0.04	-	0.03
*α*-Terpinolene	1085	1086	-	-	-	-	0.03	0.02	-	0.02	-	-
*trans*-Sabinene hydrate	1094	1098	-	0.03	0.15	0.09	0.04	-	-	0.03	0.10	-
*p*-Menth-2-en-1-ol	1114	1118	0.11	0.03	0.16	0.11	0.07	0.13	0.30	-	0.09	0.08
*p*-Menth-3-en-8-ol	1143	1145	0.46	0.03	0.13	0.05	11.98	3.10	0.11	-	0.71	0.14
Menthone	1151	1148	0.21	0.17	-	6.02	0.91	5.34	3.54	9.15	-	4.06
Isopulegol	1170	1169	-	0.02	0.06	0.13	-	-	-	0.05	0.06	0.42
*α*-Terpineol	1184	1186	0.04	0.22	0.29	1.37	1.31	0.32	3.57	2.18	1.27	1.66
Pulegone	1235	1233	30.12	1.31	25.20	26.04	5.72	24.39	19.46	30.10	34.11	57.28
Isopulegyl acetate	1274	1275	16.09	0.69	8.92	17.01	5.06	9.30	21.14	15.83	9.22	27.66
Bornyl acetate	1286	1284	0.27	0.40	-	0.55	33.24	0.29	14.15	0.53	0.10	0.12
Neo-Dihydro carveol acetate	1303	1306	-	-	-	0.03	-	0.21	-	0.04	-	0.08
Piperitenone	1341	1340	14.16	51.94	17.13	9.42	-	23.99	1.49	0.30	0.11	0.08
Piperitenone oxide	1368	1366	17.99	29.09	36.02	5.71	19.05	2.66	17.45	0.20	9.24	0.10
*trans*-Caryophyllene	1411	1417	1.39	2.18	0.72	0.70	3.54	1.27	2.51	1.48	2.75	0.70
Germacrene D	1489	1484	0.50	0.49	0.57	0.21	1.20	0.52	1.12	0.54	1.01	0.38
Bicyclogermacrene	1497	1500	0.11	0.19	0.14	0.12	0.20	0.32	0.24	-	0.29	0.39
Caryophyllene oxide	1581	1582	0.17	0.31	0.07	-	0.14	0.03	-	0.08	0.29	-
Monoterpene hydrocarbons			8.80	6.53	3.01	15.29	11.31	11.30	8.72	28.04	2.33	3.93
Oxygenated monoterpenes			80.58	89.89	94.08	82.63	82.30	72.06	85.87	68.04	82.47	93.0
Sesquiterpene hydrocarbons			2.00	2.86	1.43	1.03	4.94	2.11	3.87	2.02	4.05	1.47
Oxygenated sesquiterpenes			0.17	0.31	0.07	-	0.14	0.03	-	0.08	0.29	-
Total identified compounds			91.55	99.59	98.59	98.95	98.69	85.50	98.46	98.18	89.14	98.39
